# Association between serum neuron-specific enolase at admission and the risk of delayed neuropsychiatric sequelae in adults with carbon monoxide poisoning: A meta-analysis

**DOI:** 10.17305/bb.2024.10757

**Published:** 2024-12-01

**Authors:** Yu Zhang, Nan Gao, Yingbo Wang, Wenxin Hu, Zhihao Wang, Li Pang

**Affiliations:** 1 Department of Neurovascular Surgery, The First Hospital of Jilin University, Changchun, China; 2 Medical Quality Control Office, The Third Affiliated Hospital of Changchun University of Chinese Medicine, Changchun, China; 3 Department of Emergency, The First Hospital of Jilin University, Changchun, China; 4 Fixed Asset Management Section, The Third Affiliated Hospital of Changchun University of Chinese Medicine, Changchun, China; 5 Department of Geriatrics, Jilin Provincial Geriatric Medicine Clinical Research Center, The First Hospital of Jilin University, Changchun, China

**Keywords:** Carbon monoxide poisoning (COP), delayed neuropsychiatric sequelae (DNS), neuron-specific enolase, biomarker, meta-analysis

## Abstract

Delayed neuropsychiatric sequelae (DNS) significantly impact the quality of life in patients following acute carbon monoxide poisoning (COP). This systematic review and meta-analysis aimed to assess the relationship between serum neuron-specific enolase (NSE) levels at admission and the risk of DNS in adults after acute COP. Relevant observational studies with longitudinal follow-up were identified through searches in PubMed, Embase, Web of Science, Wanfang, and China National Knowledge Infrastructure (CNKI) databases. The random-effects model was used to aggregate results, accounting for potential heterogeneity. Nine cohort studies, including 1501 patients, were analyzed, with 254 (16.9%) developing DNS during follow-up. The pooled data indicated that elevated serum NSE in the early phase was linked to a higher risk of subsequent DNS (odds ratio per 1 ng/mL increase in NSE: 1.10, 95% confidence interval: 1.06–1.15, *P* < 0.001). Moderate heterogeneity (*I^2^* ═ 46%) among the studies was entirely attributed to one study with the longest follow-up duration (22.3 months; *I^2^* ═ 0% after excluding this study). Subgroup analyses based on country, study design, sample size, age, sex, admission carboxyhemoglobin (COHb) levels, DNS incidence, follow-up duration, and quality score yielded consistent results (*P* for subgroup differences all > 0.05). In summary, high serum NSE levels in the early phase of acute COP are associated with an increased risk of developing DNS during follow-up.

## Introduction

Carbon monoxide (CO) poisoning (COP) is the leading cause of mortality from poisoning in many countries, potentially accounting for over 50% of all fatal poisonings globally [1–3]. While patients may experience gradual improvement over several months or even up to one year, survivors of COP typically endure neurocognitive impairments associated with brain damage for years following the incident [4, 5]. Delayed neuropsychiatric sequelae (DNS) encompass a collection of neurological or cognitive disorders that manifest several weeks or months after the acute phase of COP [6]. DNS related to brain injury after COP typically manifests as impaired memory, cognitive dysfunction, depression, anxiety, and/or vestibular and motor deficits, which usually become apparent within six weeks [2]. Previous studies have reported that the occurrence of DNS in patients following acute COP ranges from 3% to 40%, and these DNS have a substantial negative impact on the patients’ quality of life and long-term clinical outcomes [7]. Consequently, it holds significant clinical importance to promptly identify individuals with acute COP who are at a heightened risk of developing DNS.

Neuron-specific enolase (NSE) is a glycolytic enzyme predominantly found in the neuronal cytoplasm of the central nervous system (CNS) [8, 9]. In humans, NSE is not naturally released into the peripheral circulation but can be detected in the blood when there is damage to the cell membrane of neuronal and glial tissue [10, 11]. As a result, the measurement of serum NSE levels has been suggested as a valuable indicator of neuronal cell damage in different clinical scenarios [12–15], including patients with acute COP [16]. However, it is still uncertain whether there is an association between serum NSE levels in the early stages and the risk of DNS in patients after acute COP. In view of this knowledge gap, the aim of this systematic review and meta-analysis was to evaluate the association between serum NSE at admission and the risk of DNS in adult patients after acute COP.

## Materials and methods

The study was planned, conducted, and reported in accordance with the Preferred Reporting Items for Systematic Reviews and Meta-Analyses (PRISMA) statement [17] and the guidelines outlined in the Cochrane Handbook [17].

### Inclusion and exclusion criteria for the studies

The inclusion criteria were developed based on PICOS recommendations and aligned with the objectives of the meta-analysis.

P (patients): Adult patients with acute COP.

I (exposure): Patients with a high serum NSE measured at presentation.

C (comparison): Patients with a low serum NSE measured at presentation.

O (outcomes): The occurrence of DNS was assessed during the follow-up period, presented as odds ratio (OR) for 1 ng/mL increment of serum NSE in the early phase, or these data could be calculated from the original studies. The diagnostic criteria for DNS in this study aligned with those used in the included studies. These criteria primarily covered psychiatric symptoms, such as depression, insomnia, and anxiety, as well as neurological symptoms including headache, dizziness, gait disturbances, cognitive impairments, and disorientation [7]. Because most patients with COP developed DNS within six weeks after CO exposure, only studies with a minimum follow-up duration of six weeks were incorporated [18].

S (study design): The observational studies with longitudinal follow-up comprised both nested case-control studies and cohort studies.

The meta-analysis excluded reviews, editorials, preclinical studies, studies involving children, cross-sectional studies, and those that did not measure serum NSE or report DNS outcomes. Additionally, studies lacking sufficient data to estimate the OR of the association between serum NSE and DNS were also excluded. When there was duplication of patient populations, priority was given to the study with the largest sample size for inclusion in the meta-analysis.

### Search of databases

We conducted electronic database searches, including PubMed, Embase, Web of Science, Wanfang, and China National Knowledge Infrastructure (CNKI), from the inception of the databases to March 28, 2024, to identify relevant studies published by that date. The search was performed using terms including (1) “neuron-specific enolase” OR “neuron specific enolase” OR “NSE;” and (2) “carbon monoxide.” Only studies of human participants published in English or Chinese as full-length articles in peer-reviewed journals were included. During our manual screening process, we examined the references cited in relevant original and review articles to identify potential relevant studies.

### Data extraction and quality evaluation

Literature searches, data collection, and evaluations of study quality were carried out independently by two authors. Any discrepancies were resolved through discussion with the corresponding author to achieve consensus. Information extracted from the included studies encompassed study characteristics, demographic variables, serum NSE measurement timing and methodologies, follow-up durations, DNS diagnostic approaches, DNS incidence, and adjustments for potential confounders when assessing the association between NSE and DNS occurrence. Quality assessment was conducted using the Newcastle–Ottawa Scale (NOS) [19], which evaluates participant selection, comparability of groups, and outcome validity. The scale comprises nine stars, with a higher number indicating higher quality.

### Ethical statement

Ethical approval was not required for this study in accordance with local/national guidelines. Written informed consent to participate in the study was not required in accordance with local/national guidelines.

### Statistical analysis

The association between baseline serum NSE levels and DNS incidence during follow-up was presented using OR and their corresponding 95% confidence intervals (CI). OR data, along with their standard errors (SE), were derived from either 95% CI or *P* values and then underwent a logarithmic transformation to stabilize variance and normalize distribution [17]. To assess between-study heterogeneity, both the Cochrane *Q* test and the *I*^2^ statistic [20] were employed. A value of *I*^2^ > 50% indicates significant heterogeneity among studies. Given this, a random-effects model was utilized to amalgamate the findings from the included studies. This model was chosen because it is recommended for accommodating potential heterogeneity and can thus yield a more generalized result [17]. To assess the impact of individual studies on the meta-analysis results, sensitivity analysis was conducted by excluding one dataset at a time [21]. Predefined subgroup analyses were conducted to ascertain the influence of study characteristics on the outcome. Cutoff values for defining subgroups were determined based on the medians of continuous variables. Publication bias was assessed using a funnel plot, which provides a visual indication of symmetry, alongside Egger’s regression asymmetry test [22]. Statistical analyses were conducted using RevMan (version 5.1; Cochrane Collaboration, Oxford, UK) and Stata software (version 12.0; Stata Corporation, College Station, TX, USA).

## Results

### Database search and study inclusion

**Figure 1.**
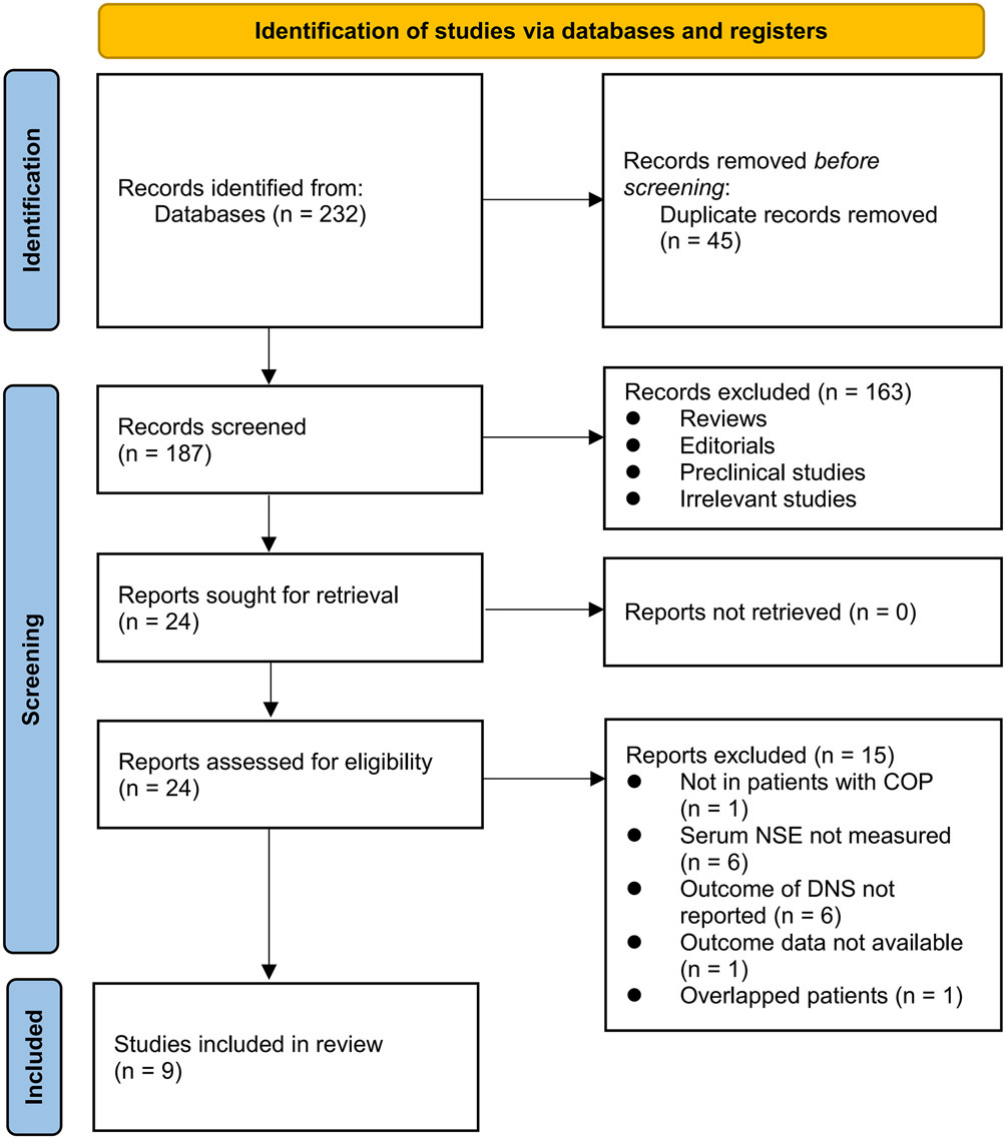
**Flowchart of database search and study inclusion.** NSE: Neuron-specific enolase; COP: Carbon monoxide poisoning; DNS: Delayed neuropsychiatric sequelae.

Figure 1 depicts the process of literature search and study retrieval. Initially, 232 records were identified from the database, and 45 duplicate entries were eliminated. Following this, 163 studies were excluded based on title and abstract screening as they did not meet the objectives of the meta-analysis. After full-text reviews of 24 studies, 15 were further excluded for reasons detailed in Figure 1. Consequently, nine studies were included for subsequent meta-analysis [23–31].

### Overview of study characteristics

The overview of the included studies is displayed in Table 1. Overall, two prospective cohort studies [28, 31] and seven retrospective cohort studies [23–27, 29, 30] were included in the meta-analysis, which were published from 2017 to 2023 and performed in China and Korea. All of the studies included adult patients with acute COP. The mean ages of the patients were 42–63 years, and the proportions of men were 43% to 73%. The mean carboxyhemoglobin (COHb) at presentation was 8.7% to 24.1%. Serum NSE was measured within 1–48 h after the presentation of the patients to emergency departments. The methods for measuring serum NSE varied among the included studies, with the Cobas Core enzyme immunoassay used in three studies [24, 28, 31], the solid-phase immunoassay used in two studies [23, 29], the immunoradiometric assay in one study [25], the enzyme-linked immunosorbent assay in one study [30], and unknown methods in another two studies [26, 27]. The serum levels of NSE of patients with and without DNS are shown in Table S1. The follow-up durations were two months in four studies [23, 29–31], three months in two studies [27, 28], six months [26], and nine months [24] in one study, and 22.3 months [25] in another study. Overall, 1501 adult patients with acute COP were included and 254 (16.9%) of them had DNS during follow-up. The details for the diagnosis of DNS in each study are also shown in Table S1. Potential confounding factors such as age, CO exposure time, and C-reactive protein (CRP) levels were adjusted to varying degrees among the included studies. The NOS of the included studies were eight or nine stars, indicating that the studies were of good quality (Table 2).

**Table 1:** Characteristics of the included cohort studies

**Study**	**Country**	**Design**	**No. of patients included**	**Mean age (years)**	**Men (%)**	**Mean COHb at admission (%)**	**Methods for NSE measuring**	**Timing of NSE measuring**	**Follow-up time for DNS after discharge**	**Diagnosis of DNS**	**No. of patients with DNS**	**Variables adjusted**
Cao, 2017	China	RC	56	62.7	48.2	22.3	Solid-phase immunoassay	Within 24h of presentation to ED	2 months	Clinical evaluation	20	Age, coma, time from CO exposure to admission, CK-MB, CRP, and LDH
Moon, 2018	Korea	RC	236	45.4	72.9	10.1	Immunoradiometric assay	At presentation to ED	22.3 months	Clinical evaluation	20	Age, initial GCS, CRP, initial TnI, and arterial HCO3
Cha, 2018	Korea	RC	98	56	60	19	Cobas Core enzyme immunoassay	Within 2h after ED arrival	9 months	Clinical evaluation	8	Age, CO exposure time, GCS, loss of consciousness, CK, and TnI
Jung, 2019	Korea	RC	432	55	67.4	8.7	NR	Within 1h after ED arrival	6 months	Clinical evaluation	68	Age, CO exposure time, GCS, lactate, and CK
Li, 2019	China	RC	189	43	43	20	NR	Within 48h of presentation to ED	3 months	Clinical evaluation	37	Age, coma, brain MRI abnormality at admission, and HIF-1a genotype
Nah, 2021	Korea	PC	187	42	68.5	11.7	Cobas Core enzyme immunoassay	At 48h of presentation to ED	3 months	Clinical evaluation	25	Age, initial GCS, and CRP
Sun, 2021	China	RC	79	54.6	59.5	23.1	Solid-phase immunoassay	Within 24h of presentation to ED	2 months	Clinical evaluation	34	Age, coma, CO exposure time, lactate, CK, CK-MB, CRP, LDH, and ADC value of globus pallidum on cerebral MRI
Yang, 2022	China	RC	69	54.1	60.8	15.5	ELISA	At presentation to ED	2 months	Clinical evaluation	27	Age, CO exposure time, and S100β
Liang, 2023	China	PC	155	55.1	54.8	24.1	Cobas Core enzyme immunoassay	At presentation to ED	2 months	Clinical evaluation	15	Age, sex, history of stroke/TIA, and duration of coma

COHb: Carboxyhemoglobin; NSE: Neuron-specific enolase; DNS: Delayed neuropsychiatric sequelae; RC: Retrospective cohort; PC: Prospective cohort; NR: Not reported; ELISA: Enzyme-linked immunosorbent assay; ED: Emergency department; COP: Carbon monoxide poisoning; CO: Carbon monoxide; CRP: C-reactive protein; CK: Creatine kinase; CK-MB: Creatine kinase myocardial band; LDH: Lactate dehydrogenase; GCS: Glasgow Coma scale; HCO3: Bicarbonate; TnI: Troponin I; MRI: Magnetic resonance imaging; HIF-1a: Hypoxia-induced factor 1a; ADC: Apparent diffusion coefficient.

**Table 2:** Quality evaluation of the included cohort studies via the Newcastle–Ottawa Scale

**Study**	**Representativeness of the exposed cohort**	**Selection of the non-exposed cohort**	**Ascertainment of exposure**	**Outcome not present at baseline**	**Control for age**	**Control for other confounding factors**	**Assessment of outcome**	**Long enough follow-up duration**	**Adequacy of follow-up of cohorts**	**Total**
Cao, 2017	1	1	1	1	1	1	1	1	1	9
Moon, 2018	0	1	1	1	1	1	1	1	1	8
Cha, 2018	1	1	1	1	1	1	1	1	1	9
Jung, 2019	1	1	1	1	1	1	1	1	1	9
Li, 2019	0	1	1	1	1	1	1	1	1	8
Nah, 2021	1	1	1	1	1	1	1	1	1	9
Sun, 2021	1	1	1	1	1	1	1	1	1	9
Yang, 2022	0	1	1	1	1	1	1	1	1	8
Liang, 2023	1	1	1	1	1	1	1	1	1	9

### Meta-analysis results

**Figure 2.**
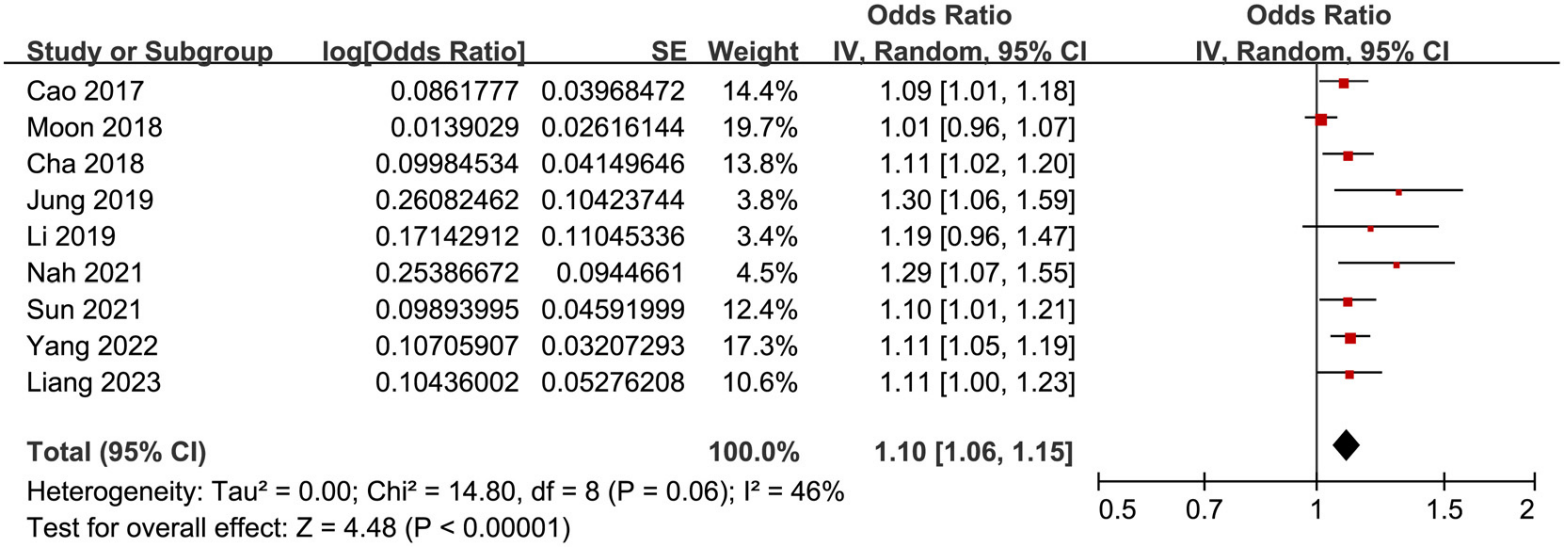
**Forest plots for the meta-analyses regarding the association between NSE at early phase and the incidence of DNS during follow-up in adult patients with acute COP.** NSE: Neuron-specific enolase; DNS: Delayed neuropsychiatric sequelae; COP: Carbon monoxide poisoning.

Pooled results showed that serum NSE in the early phase was associated with a higher risk of DNS in adult patients of acute COP during follow-up (OR for per 1 ng/mL increment of NSE: 1.10, 95% CI: 1.06–1.15, *P* < 0.001; Figure 2) with moderate heterogeneity (*I*^2^ ═ 46%). Sensitivity analysis by excluding one study at a time showed consistent results (OR: 1.09–1.12, *P* all < 0.001; Table 3). Specifically, excluding the study by Moon et al. [25] adequately diminished between-study heterogeneity (*I*^2^ ═ 0%, Table 3). Further subgroup analyses according to study country, design, sample size, mean age, proportion of men, COHb at admission, incidence of DNS, follow-up duration, and quality score also showed similar results (*P* for subgroup difference all > 0.05; Table 4).

**Table 3:** Results of sensitivity analysis by excluding one study at a time

**Study omitted**	**OR (95% CI)**	***P* for effect**	***I* ^2^**
Cao, 2017	1.11 (1.05, 1.17)	<0.001	53%
Moon, 2018	1.12 (1.08, 1.16)	<0.001	0%
Cha, 2018	1.11 (1.05, 1.16)	<0.001	52%
Jung, 2019	1.09 (1.05, 1.14)	<0.001	41%
Li, 2019	1.10 (1.05, 1.15)	<0.001	50%
Nah, 2021	1.09 (1.05, 1.14)	<0.001	39%
Sun, 2021	1.11 (1.05, 1.16)	<0.001	52%
Yang, 2022	1.10 (1.05, 1.16)	<0.001	50%
Liang, 2023	1.10 (1.05, 1.16)	<0.001	52%

OR: Odds ratio; CI: Confidence interval.

**Table 4:** Subgroup analyses for the association between NSE at presentation and the incidence of DNS in patients with COP

**Study characteristics**	**Datasets number**	**OR (95% CI)**	***I* ^2^**	***P* for subgroup effect**	***P* for subgroup difference**
Country					
China	5	1.11 (1.06, 1.15)	0%	<0.001	
Korea	4	1.13 (1.02, 1.26)	75%	0.02	0.72
Design					
Prospective	2	1.17 (1.02, 1.35)	48%	0.03	
Retrospective	7	1.09 (1.04, 1.14)	46%	<0.001	0.35
Sample size					
<100	4	1.10 (1.06, 1.15)	0%	<0.001	
≥100	5	1.14 (1.03, 1.27)	69%	0.01	0.55
Mean age					
<55 years	5	1.10 (1.03, 1.18)	63%	0.006	
≥55 years	4	1.11 (1.06, 1.16)	0%	<0.001	0.86
Men					
< 60%	5	1.07 (1.02, 1.12)	33%	0.005	
≥ 60%	4	1.14 (1.07, 1.22)	29%	<0.001	0.12
Mean COHb at admission					
<20%	5	1.12 (1.03, 1.20)	71%	0.004	
≥20%	4	1.10 (1.05, 1.16)	0%	<0.001	0.82
Incidence of DNS					
< 15%	4	1.09 (1.01, 1.19)	66%	0.03	
≥ 15%	5	1.11 (1.07, 1.16)	0%	<0.001	0.70
Follow-up duration					
2 or 3 months	6	1.11 (1.07, 1.16)	0%	<0.001	
6 months or longer	3	1.09 (1.01, 1.21)	73%	0.04	0.73
Quality score					
NOS ═ 8	3	1.07 (0.99, 1.17)	68%	0.04	
NOS ═ 9	6	1.12 (1.07, 1.16)	0%	<0.001	0.41

NSE: Neuron-specific enolase; DNS: Delayed neuropsychiatric sequelae; COP: Carbon monoxide poisoning; OR: Odds ratio; CI: Confidence interval; COHb: Carboxyhemoglobin; NOS: Newcastle–Ottawa Scale.

### Publication bias

**Figure 3.**
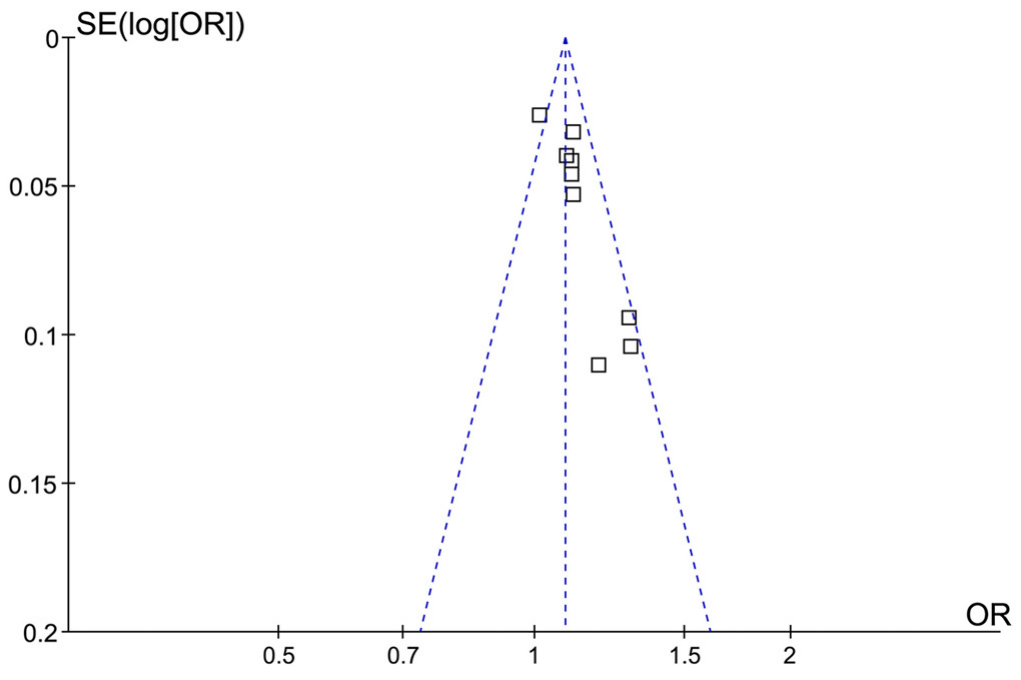
**Funnel plots for the publication bias underlying the meta-analysis regarding the association between NSE and the incidence of DNS.** NSE: Neuron-specific enolase; DNS: Delayed neuropsychiatric sequelae.

Figure 3 displays the funnel plots for the meta-analysis examining the association between baseline serum NSE levels and DNS incidence. Visual inspection suggests symmetry in the plots, indicating low publication bias. Furthermore, Egger’s regression tests yielded a *P* value of 0.18, indicating a low likelihood of publication bias.

## Discussion

This study integrated findings from nine cohort studies, revealing a correlation between higher serum NSE levels in the early phase and an elevated risk of DNS in adults with acute COP. Sensitivity analysis, which involved excluding one study at a time, consistently upheld these results. Specifically, excluding the study by Moon et al. [25] significantly reduced the level of between-study heterogeneity. In addition, further subgroup analyses confirmed that the results were not significantly affected by study characteristics such as study country, design, sample size, age, sex, COHb at admission, incidence of DNS, follow-up duration, or study quality scores. Collectively, these findings imply that elevated serum NSE levels upon presentation may be associated with a higher incidence of DNS in adult patients with acute COP.

To our knowledge, this study may represent the first meta-analysis to comprehensively summarize the current understanding of the association between serum NSE levels and the risk of DNS in adult patients with acute COP. Several methodological strengths warrant attention prior to interpreting the meta-analysis results. Firstly, an extensive literature search was conducted across five commonly used English and Chinese electronic databases, ensuring access to the most up-to-date literature on the topic. Secondly, all included studies were cohort studies, suggesting a longitudinal relationship between elevated serum NSE levels and an increased risk of DNS in these patients. Thirdly, multivariate regression analyses were employed in all included studies to evaluate the association between serum NSE and subsequent DNS risk, demonstrating potential independence from study characteristics, such as age, duration of CO exposure, and systemic inflammation indicated by serum CRP levels. This is important because systemic inflammation has been recognized as a potential mechanism underlying the pathogenesis of DNS after acute COP [32]. Finally, multiple sensitivity and subgroup analyses suggested the robustness of the finding, which was not primarily driven by any single included studies and was unlikely to be significantly modified by a series of predefined study characteristics. Because methods for measuring NSE are mature and ready to use in most clinical centers, these findings support incorporating serum NSE as a predictor for DNS when managing patients with acute COP.

Moderate heterogeneity was observed among the included studies, which was diminished after excluding the study by Wang et al. [33], as evidenced by the results of the sensitivity analysis. These findings suggest that this study contributed significantly to the between-study heterogeneity. Compared to other studies, the follow-up duration of the study by Moon et al. was much longer (22.3 vs 2∼9 months). Due to the reliance on symptom clusters for the diagnosis of DNS following acute COP, various clinical factors may contribute to the manifestation of these neuropsychiatric symptoms, in addition to acute COP-related brain injury as the follow-up duration increases. This may explain the lack of a significant association between NSE at the early phase and the risk of DNS observed in the study by Wang et al. [33]. However, considering that the majority of DNS patients experience relevant symptoms within six weeks after acute COP, the predictive value of NSE for long-term neuropsychiatric symptoms is of lesser significance [34]. From a physiological standpoint, NSE is primarily situated within the cytoplasm of neurons in the CNS, with minimal presence in human blood. However, the release of NSE into the cerebrospinal fluid and bloodstream can occur due to various stimuli or toxic agents [35]. The findings of this meta-analysis further support the potential clinical utility of NSE as a biomarker for predicting the likelihood of developing DNS following acute COP.

This study presents several limitations. Firstly, the inclusion of a limited number of studies in the meta-analysis, with generally small sample sizes, may impact the robustness of the findings. Additionally, seven of the studies have a retrospective design, which could introduce recall and selection biases. Therefore, it is recommended that the results of the meta-analysis be validated in large-scale prospective studies. Only studies published in English and Chinese were included, which may lead to biases in the meta-analysis. Moreover, the included studies are all from China and Korea, and studies from other countries are needed to determine if the association between NSE and DNS remains consistent in patients of other ethnicities. Furthermore, future studies should aim to determine the optimal measuring methods, timing, and cutoff values of serum NSE for predicting the risk of DNS following acute COP. Lastly, while multivariate analyses were utilized in all included studies to assess the association between NSE and DNS, there remains the possibility of residual confounding factors that were not accounted for.

## Conclusion

In summary, the meta-analysis results suggest a possible connection between elevated serum NSE levels early on and a heightened risk of DNS in adult patients post-acute COP. Rigorous prospective studies on a larger scale are essential to confirm these findings. Nonetheless, these results strengthen the argument for considering serum NSE measurement as a predictive tool for DNS in acute COP patients.

## Supplemental data

**Table S1:** Levels of NSE and definitions of DNS in each study

**Study**	**Serum NSE of patients with DNS (ng/mL)**	**Serum NSE of patients without DNS (ng/mL)**	**Methods for the diagnosis of DNS**
Cao, 2017	19.12 ± 5.64	11.38 ± 4.12	Patients with acute COP who experienced a period of lucidity followed by the emergence of neuropsychiatric symptoms such as cognitive impairment, increased muscle tone, and urinary and fecal incontinence were diagnosed as DNS. This diagnosis could be confirmed with cranial CT or MRI showing extensive white matter damage. The diagnosis of DNS was achieved via clinical interview by trained medical professionals.
Moon, 2018	16.8 (12.6–24.5)^a^	15.5 (10.8–23.0)^a^	Dependency on assistance with daily activities (scores of 1–3 on the GOS) that developed after a lucid interval was designated DNS. Because the lucid interval reportedly ranged from 2 to 45 days, DNS must have developed within 3 months after COP. The diagnosis of DNS was achieved via clinical follow-up by two blinded physicians.
Cha, 2018	45.6 (23.1–53.9)^a^	21.5 (16.0–27.3)^a^	DNS was diagnosed as various neuropsychiatric symptoms and signs after apparent recovery from acute COP, which included mental deterioration, cognitive dysfunction, amnesia, gait disturbance, mutism, urinary or fecal incontinence, psychosis, depression, and Parkinsonism. The diagnosis of DNS was achieved via clinical follow-up.
Jung, 2019	43.3 ± 51.1	16.1 ± 17.2	DNS was diagnosed according to the neurological outcome measured at 6-month post-admission via the Cerebral Performance Category scale of 3∼5 points.
Li, 2019	17.57 ± 3.45	9.95 ± 1.93	Patients with acute COP who experienced a period of lucidity followed by the emergence of neuropsychiatric symptoms, which included: (1) psychiatric and consciousness disorders (dementia, stupor, delirium, or decorticate state); (2) extrapyramidal disorders (manifestations of parkinsonian syndrome); (3) pyramidal tract damage (hemiplegia, positive pathological reflexes, or urinary and fecal incontinence); (4) focal cortical dysfunction (such as aphasia, blindness, or secondary epilepsy). CT or MRI could reveal symmetrical lesions in the bilateral globus pallidus and extensive demyelination in the cerebral white matter. EEG could show moderate to severe diffuse abnormalities. The diagnosis of DNS was achieved via clinical follow-up by trained medical professionals.
Nah, 2021	23.02 (17.5–27.96)^a^	18.19 (14.46–23.87)^a^	DNS were diagnosed as neurological abnormalities developing within 3 months after discharge and included symptoms such as depression, psychosis, urinary or fecal incontinence, disturbed consciousness, mutism, gait disturbance, memory disorder, and Parkinson-like syndrome. The diagnosis of DNS was achieved by clinical interview with the psychiatrists.
Sun, 2021	18.73 ± 5.46	11.98 ± 4.16	DNS was diagnosed as a group of neuropsychological disorders that occured after a transient improvement or a symptom-free interval of acute COP. These acute symptoms included memory loss, motor dysfunction, mental behavior abnormality, disturbance of intelligence, and bladder/bowel dysfunction after a latent period of pseudo-recovery for 2 days to 1 year. The diagnosis of DNS was achieved by clinical interview at 60 days after COP with trained medical professionals.
Yang, 2022	14.83 ± 3.81	11.05 ± 2.90	DNS was diagnosed as various neuropsychiatric symptoms and signs after apparent recovery from acute COP, which included mental deterioration, cognitive dysfunction, amnesia, gait disturbance, mutism, urinary or fecal incontinence, psychosis, depression, and Parkinsonism. The diagnosis of DNS was achieved by clinical interview at 60 days after COP with trained medical professionals.
Liang, 2023	113.7 ± 54.8	25.8 ± 13.0	DNS was diagnosed as a series of neurological and psychiatric symptoms after apparent recovery from acute COP, including mental disturbances, slow responses, cognitive dysfunction, gatism, dystonia, and even dementia. The diagnosis of DNS was achieved by clinical interview with physicians.

^a^Presented as medians and interquartile ranges. NSE: Neuron-specific enolase; DNS: Delayed neuropsychiatric sequelae; COP: Carbon monoxide poisoning;

## Data Availability

All the data generated during the study are within the manuscript.
